# IgA Plasma Cells Are Long-Lived Residents of Gut and Bone Marrow That Express Isotype- and Tissue-Specific Gene Expression Patterns

**DOI:** 10.3389/fimmu.2021.791095

**Published:** 2021-12-24

**Authors:** Joel R. Wilmore, Brian T. Gaudette, Daniela Gómez Atria, Rebecca L. Rosenthal, Sarah Kim Reiser, Wenzhao Meng, Aaron M. Rosenfeld, Eline T. Luning Prak, David Allman

**Affiliations:** ^1^ The Department of Pathology and Laboratory Medicine, Perelman School of Medicine at the University of Pennsylvania, Philadelphia, PA, United States; ^2^ Department of Microbiology and Immunology, State University of New York (SUNY) Upstate Medical University, Syracuse, NY, United States

**Keywords:** plasma cell, isotype, antibody, gut, IgA, bone marrow

## Abstract

Antibody secreting plasma cells are made in response to a variety of pathogenic and commensal microbes. While all plasma cells express a core gene transcription program that allows them to secrete large quantities of immunoglobulin, unique transcriptional profiles are linked to plasma cells expressing different antibody isotypes. IgA expressing plasma cells are generally thought of as short-lived in mucosal tissues and they have been understudied in systemic sites like the bone marrow. We find that IgA^+^ plasma cells in both the small intestine lamina propria and the bone marrow are long-lived and transcriptionally related compared to IgG and IgM expressing bone marrow plasma cells. IgA^+^ plasma cells show signs of shared clonality between the gut and bone marrow, but they do not recirculate at a significant rate and are found within bone marrow plasma cells niches. These data suggest that systemic and mucosal IgA^+^ plasma cells are from a common source, but they do not migrate between tissues. However, comparison of the plasma cells from the small intestine lamina propria to the bone marrow demonstrate a tissue specific gene transcription program. Understanding how these tissue specific gene networks are regulated in plasma cells could lead to increased understanding of the induction of mucosal versus systemic antibody responses and improve vaccine design.

## Introduction

Newly generated plasma cells either die within days, or take up residency in tissues such as the bone marrow (BM) or the lamina propria of the small intestine (siLP) where they can persist indefinitely as high output antibody secreting cells (1–4). Whereas the induction and maintenance of IgG-secreting cells in BM has been studied extensively (5), much less is known about the factors that influence the induction and maintenance of IgA-secreting plasma cells in the gut or other tissues. IgA is the dominant antibody class produced throughout the body and the continuous secretion of IgA along the intestinal epithelium is critical for preventing intestinal disequilibrium and shaping communities of commensal microbes (6). Furthermore, recent data reveal that commensal bacteria in the gut and potentially elsewhere can induce the production of serum IgA and IgG antibodies that form a protective humoral barrier against systemic infection (7, 8). Consistent with this function, many microbiota-induced IgA-secreting plasma cells can be identified in non-mucosal sites such as the BM (7) and spleen (9). It is not understood how plasma cells that arise in mucosal tissues home to the BM, or whether such cells persist for extended periods as marrow-resident cells.

It is widely accepted that the BM contains unique regulatory microenvironments needed for long-term plasma cell survival. Consistent with this view, BM plasma cells localize preferentially in perivascular niches orchestrated by CXCL12-positive stromal cells where they interact with several diverse cell types that may contribute to plasma cell survival (5). However, unlike IgM- and IgG-secreting plasma cells in the spleen, lymph node, and BM, intestinal IgA^+^ plasma cells home to and remain in mucosal tissues by employing the chemokine receptors CCR9, CCR10, and the integrin α_4_β_7_ (10, 11). Whether these processes are used by IgA-secreting plasma cells in BM is uncertain.

It has also been proposed that plasma cells have function beyond antibody generation (12). Plasma cells in the central nervous system and other sites produce immunosuppressive cytokines such as IL-10 (13), and many IgA-secreting cells in the gut produce the antimicrobial mediators tumor-necrosis factor-α and inducible nitric oxide synthase (14). Thus, subpopulations of plasma cells may activate unique gene expression modules needed for additional immunomodulatory functions. Consistent with this idea, it was shown recently that plasma cells expressing different immunoglobulin (Ig) heavy chain (IgH) isotypes have distinct gene expression profiles (15). However, it is unknown whether IgA-secreting cells in the BM and siLP maintain distinct, tissue-associated transcriptional profiles, or are dictated by IgH isotype alone. Furthermore, it is becoming clear that mature IgM- and IgA-secreting plasma cells often retain functional Ig surface receptors (16), raising additional questions about the impact of particular IgH isotypes on plasma cell gene expression and function.

Based on these considerations we sought to ask: 1) Are BM IgA-secreting plasma cells long-lived BM-resident cells? If so, do BM IgA-secreting plasma cells localize to specialized microenvironments within the BM parenchyma that overlap with those employed by IgG-secreting cells? 2) Does movement of IgA^+^ plasma cells to the BM coincide with the expression of unique gene expression patterns, or do BM IgA^+^ plasma cells share gut-associated gene expression patterns with IgA^+^ plasma cells in the siLP? And 3) To what extent does IgA-correlated gene expression require surface IgA expression? Our results demonstrate that BM IgA^+^ plasma cells are long-lived and readily integrate into canonical perivascular plasma cell niches where they express a unique IgA-correlated gene expression program mirroring their counterparts in the gut. Furthermore, we show that the gut environment also imparts tissue-specific gene expression on siLP plasma cells, regardless of surface Ig isotype.

## Methods

### Animals

B6.Blimp1^+/GFP^ (17), B6.IgA^-/-^ (18), and C57BL/6 (B6) mice were bred and maintained at the University of Pennsylvania under specific pathogen free conditions. All experiments were performed in accordance with the Office of Regulatory Affairs Institutional Animal Care and Use Committee.

### Flow Cytometry and Cell Sorting

Cells were stained for flow cytometric analysis as previously described (19). Exclusion of dead cells was performed using Zombie Aqua Fixable Viability kit (BioLegend) according to the manufacturer’s instructions. The following antibodies were used for staining and analysis: PE-anti-CD138 (BD; 281-2), -CxCR4 (eBioscience (eBio); 2B11), -IgM (BD; R26-46), PerCP-eFluor710-anti-IgM (eBio; II/41), -CCR9 (eBio; eBioCW-1.2), PE-Cy7-anti-CD4 (eBio; GK1.5), -CD8a (eBio; 53-6.7), -Gr1 (eBio; RB6-8C5), -F4/80 (eBio; BM8), -TER-119 (BioLegend), APC-anti-B220 (Tonbo; RA3-6B2), APC-Cy7-anti-IgD (BioLegend; 11-26c.2a), Biotin-anti-IgA (BioLegend; RMA-1), -α_4_β_7_ (BioLegend; DATK32), BrilliantViolet605-anti-Thy1.2 (BioLegend; 53-2.1), AF700 anti-CD45.2 (BioLegend; 104), and BV785-anti-CD19 (BioLegend; 6D5). To detect biotin-conjugated antibodies we used streptavidin-BV421 (BioLegend). Labeling with 5-bromo-2’-deoxyuridine was performed by adding 0.6mg/ml BrdU and 1mg/ml sucrose into the drinking water. Cells were then stained for flow cytometry with the methods described above. Following the last wash step the cells were fixed and permeabilized using Fixation Medium A and Permeabilization Medium B (ThermoFisher). Cells were then treated with a DNase solution containing 0.3M NaCl, 4mM MgCl_2_, and 0.25mg/ml DNaseI (Sigma) for 40 minutes, and then washed and stained with APC-anti-BrdU (eBio; BU20A). Data was collected using an LSRII Fortessa (BD) and cell sorting was performed on an AriaII Cell Sorter (BD). All cell sorting experiments were performed with double sorting, bone marrow plasma cells were first sorted on plasma cell identity (Live, Dump^-^ (CD4, CD8, F4/80, and Ter119), IgD^-^, CD138^+^, Blimp^GFP+^) then sorted again for plasma cell identity with the addition of antibody isotype (IgA^+^, IgM^+^, or IgA^-^IgM^-^) and siLP plasma cells were sorted without CD138 and on IgA^+^ for both sorts (IgA^-/-^ samples were double sorted on Blimp^GFP+^ without the use of IgA).

### Bone Marrow Imaging

We followed the tissue clearing and staining protocol reported by Acar et al. (20). Bones from B6.Blimp1^+/GFP^ reporter mice were fixed in 4% PFA for 6 h, immersed in 30% sucrose O/N, cryopreserved in OCT (Fisher Scientific) and longitudinally bisected on a cryostat. For staining, half bones were rinsed in PBS and blocked overnight in staining solution (10% DMSO, 0.5% IgePal630 and 5% donkey serum) with TrueStainFcX FcR block (Biolegend). The same staining solution was used with labeled antibodies against GFP (polyclonal rabbit anti-mouse, Cat#A21311, Life Technologies), IgA (clone RMA-1, Biolegend), IgM (BV421 clone RMM-1, Biolegend), and Laminin 1 + 2 (polyclonal rabbit anti-mouse, Cat# ab7463, Abcam) for 3 days, followed by PBS wash for 1 day. Anti-laminin and anti-IgA antibodies were conjugated to CF568 and CF647 respectively using a Biotium Mix-n-Stain antibody labeling kit according to manufacturer’s protocols. To clear the tissue, bones were dehydrated in increasing ethanol concentration and then incubated overnight in BABB solution (1:2, benzyl alcohol: benzyl benzoate). Images were obtained on a Zeiss LSM710 confocal microscope and analyzed using Imaris 8.2 (Bitplane) software.

### Parabiosis Surgery

C57BL/6 (CD45.2) and B6.CD45.1 adult female mice were connected by parabiosis as previously described (21). In brief, mice were anesthetized with isoflurane and/or ketamine (100-200 mg/kg) and xylazine (5-16 mg/kg). A skin incision was made from the olecranon to the knee of each of the mice to be joined. The elbows and knees of the two paired mice were then tied together with surgical suture, followed by connecting of the skin with surgical sutures and staples. For pain control, mice were given buprenorphine (0.1 mg/kg) and ketoprofen (5 mg/kg) for up to 3 days after surgery. Mice were provided with sulfamexathole (400 mg/L) and trimethoprim (80 mg/L) antibiotics in their drinking water to prevent infection. Mice were monitored for signs of pain, infection, or damage to sutures. Blood was periodically drawn to check for anastomoses, which appeared complete by day 17 at which time mice were euthanized and the BM and siLP were harvested.

### ELISpot Assays

ELISpot assays were performed according to standard protocols. Briefly, ELISpot plates (EMD Millipore MSIPS4W10) were coated with anti-mouse Ig(H+L) (Southern Biotech 1010-01) in a sodium carbonate/bicarbonate solution pH 9.6 and detection was performed with anti- IgM, IgA, IgG, κ, and λ antibodies conjugated to biotin (Southern Biotech). Biotinylated antibodies were revealed with streptavidin-alkaline phosphatase (Sigma E2636) and developed with BCIP/NBT (Sigma B1911). ELISpot plates were imaged and spots were counted using a CTL Immunospot Analyzer (Cellular Technologies Limited).

### V_H_D_H_J_H_ Sequencing

Amplicons encoding IgA from siLP and BM plasma cells were generated using the semi-nested PCR approach and oligonucleotides reported by Lindner et al. (22). However oligonucleotides were modified to include adaptor sequences for the Illumina NexteraXT kit and are as follows: Promiscuous V_H_ Primer Pool, Primer #1- 5’-GTC TCG TGG GCT CGG AGA TGT GTA TAA GAG ACA GNG AGG TGC AGC TGC AGG AGT CTG G-3’, #2- 5’-GTC TCG TGG GCT CGG AGA TGT GTA TAA GAG ACA GTN GAG GTG CAG CTG CAG GAG TCT GG-3’, #3- 5’-GTC TCG TGG GCT CGG AGA TGT GTA TAA GAG ACA GTG NGA GGT GCA GCT GCA GGA GTC TGG-3’, C_α_ Primer Pool #1- 5’-TCG TCG GCA GCG TCA GAT GTG TAT AAG AGA CAG NGA GCT CGT GGG AGT GTC AGT G-3’, #2- 5’-TCG TCG GCA GCG TCA GAT GTG TAT AAG AGA CAG ANG AGC TCG TGG GAG TGT CAG TG-3’, #3- 5’-TCG TCG GCA GCG TCA GAT GTG TAT AAG AGA CAG TAN GAG CTC GTG GGA GTG TCA GTG-3’. PCR products were purified by gel electrophoresis and extraction (QIAquick Gel Extraction kit, Qiagen), and DNA concentrations determined with a Qubit fluorometer (Invitrogen). Libraries were then loaded onto an Illumina MiSeq in the Human Immunology Core Facility at the University of Pennsylvania. 2 x 300 bp paired end kits were used for all experiments (Illumina MiSeq Reagent Kit v3, 600 cycle, Illumina Inc., San Diego, Cat. No. MS-102-3003).

### V_H_D_H_J_H_ Sequence Analysis

Raw sequencing data in FASTQ format were quality controlled using pRESTO version 0.5.10 (23) as described in (24). Briefly, paired reads were aligned, low quality reads were removed (those with an average Phred quality score of < 30), individual nucleotides with a quality score < 30 were masked with *N*s, and sequences with more than 10 *N*s were removed.

The remaining sequences were aligned and annotated with V & J genes using IgBLAST version 1.16.0 (25). Following this, sequences were imported into ImmuneDB (26) version 0.29.10 for clonal inference and downstream analysis. Sequences sharing the same V-gene, J-gene, and CDR3 length were hierarchically clustered and groups of sequences sharing at least 85% amino-acid homology in the CDR3 region were grouped into clones. The framework 1 region of VH genes was trimmed off to remove the primer binding region to ensure the accuracy of SHM calculation.

### RNA-Sequencing

Cell populations were sorted directly into Trizol with 0.5% 2-ME and held at -80°C until RNA preparation. RNA was prepared using Trizol RNA-extraction according to the manufacturer’s protocol (Thermo-Fisher Scientific). RNA was co-precipitated using glycogen as a carrier. RNA was quantified using Qubit RNA high sensitivity fluorometric assay. cDNA was prepared using Takara Clontech SMART-Seq^®^ HT Kit according to protocol using 500-1000 ng RNA as input. cDNA was quantified and qualified using the HS-DNA assay on an Agilent 4200 Tapestation. RNA-seq libraries were constructed using the Illumina Nextera XT kit with 150 ng cDNA input. Libraries were quality controlled and quantified by Tapestation and pooled at equal molar ratio prior to sequencing on Illumina Nextseq500 (75bp SE v2) machines.

### Pseudoalignment and Gene Expression

Transcript abundance was computed by pseudoalignment with Kallisto (27). Transcript per million (TPM) values were then normalized and fitted to a linear model by empirical Bayes method with the Voom and Limma R packages (28, 29). Differential gene expression was defined as a Benjamini-Hochberg corrected p-value of < 0.05 and fold change > 2, unless otherwise noted.

### Gene Ontology Cluster Enrichment

GO analysis was performed using the DAVID Bioinformatics Resources 6.8, NIAID, NIH as well as ClueGO and Cytoscape. The enrichment score calculation from Bonferroni step-down adjusted P values < 0.05 was used to define enriched terms and clusters of terms, choosing the term with highest number of represented genes as founder term for each cluster. Non-descriptive or overly general terms were disregarded in favor of the term with the next highest number of genes represented (30–32).

## Results

### Long-Lived IgA-Secreting Cells Dominate the BM and siLP Plasma Cell Pools

One proposed model of antibody mediated homeostasis in the gut posits that IgA^+^ plasma cell responses in the siLP are designed to allow for the adaptation to changing microbial communities in the gut over time, resulting in displacement of long-lived plasma cells with newcomers (33). Recent work from our group and others describing large numbers of BM IgA^+^ plasma cells under certain conditions led us to ask if the BM plasma cell niche was capable of inducing long-term survival of IgA^+^ plasma cells (7, 34). To characterize plasma cells longevity, we developed a flow cytometric approach to distinguish BM plasma cells secreting each heavy chain isotype. To ensure that our analyses focused on *bona fide* plasma cells we employed B6.Blimp1^+/GFP^ adult mice born and raised in our colony, focusing on viable GFP/Blimp1^+^ CD138^+^ BM cells defined further by surface expression of IgM or IgA. Notably, although activated B cells are thought to down-regulate surface Ig expression during plasma cell differentiation, consistent with recent studies (16, 35) we readily identified surface IgM^+^ and IgA^+^ cells among intact Blimp1^+^ CD138^+^ cells (**Figure S1A**). By contrast, we were unable to discern surface IgG expression among any GFP/Blimp1^+^ CD138^+^ BM cells. To confirm that each population was highly enriched for cells secreting the expected Ig heavy chain isotype, we sorted cells from each GFP/Blimp1^+^ CD138^+^ fraction into ELISpot plates coated with antibodies specific for total Ig(H+L), and detected with isotype-specific antibodies. As expected, IgA-secreting cells were readily and exclusively detected among the IgA^+^ fraction, while the IgM^+^ fraction was enriched only for IgM-secreting cells, and the IgA^-^ IgM^-^ fraction contained only IgG-secreting cells (**Figures S1B, C**).

**Figure 1:**
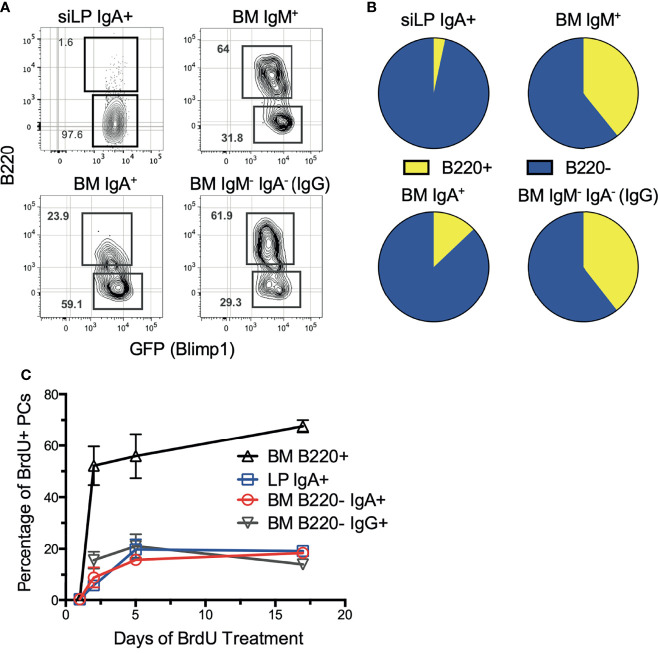
IgA^+^ plasma cells are long-lived in the BM and siLP. Under homeostatic conditions, B6.Blimp1^+/GFP^ mice were euthanized, then siLP and BM cells were analyzed by flow cytometry to determine the degree of surface B220 expression on plasma cells of each isotype. **(A)** Shown are representative images of viable, Dump^-^, IgD^-^, CD138^+^, Blimp-^GFP+^ plasma cells that were further differentiated by surface immunoglobulin isotype and then analyzed for frequency of B220 expression. **(B)** Graphical representation of data in **(A)** compiling data from 4 mice/group. **(C)** Mice were treated with BrdU in their drinking water continuously until euthanasia for 0, 1, 5, and 17 days. BM and siLP cells were then stained for plasma cell isotypes as in **(A)**, followed by intracellular staining with an anti-BrdU antibody. Each experiment used 4 mice per group and is representative of 2-4 independent experiments.

Previously we described surface B220 expression as a defining feature of short-lived plasma cells in the spleen and BM (36). Therefore, we sought to define the phenotype of each BM plasma cell isotype based on B220 expression. Staining revealed that the majority of BM IgA^+^ plasma cells were B220^-^, consistent with a long-lived phenotype. In contrast, the majority of IgM^+^ and IgM^-^IgA^-^ plasma cells were B220^+^ (**Figures 1A, B** **)**. Interestingly, the siLP IgA^+^ plasma cell pool consists almost entirely of IgA^+^B220^-^ plasma cells, indicative of a long-lived phenotype. These findings contrast with reports of siLP plasma cell pools being dominated by short-lived cells (33, 37). To confirm these results, we utilized continuous *in vivo* BrdU labeling to assess population turnover kinetics. As shown, BM and siLP IgA^+^B220^-^ plasma cells incorporated relatively little BrdU over 17 days compared to B220^+^ BM plasma cells, but a similar amount to IgA^-^B220^-^ BM plasma cells. (**Figure 1C**). Our data suggest that mouse BM and siLP can harbor substantial numbers of long-lived IgA-secreting plasma cells.

### IgA^+^ Plasma Cells Are Non-Circulating Residents of the BM and siLP Plasma Cell Pools

**Figure 2:**
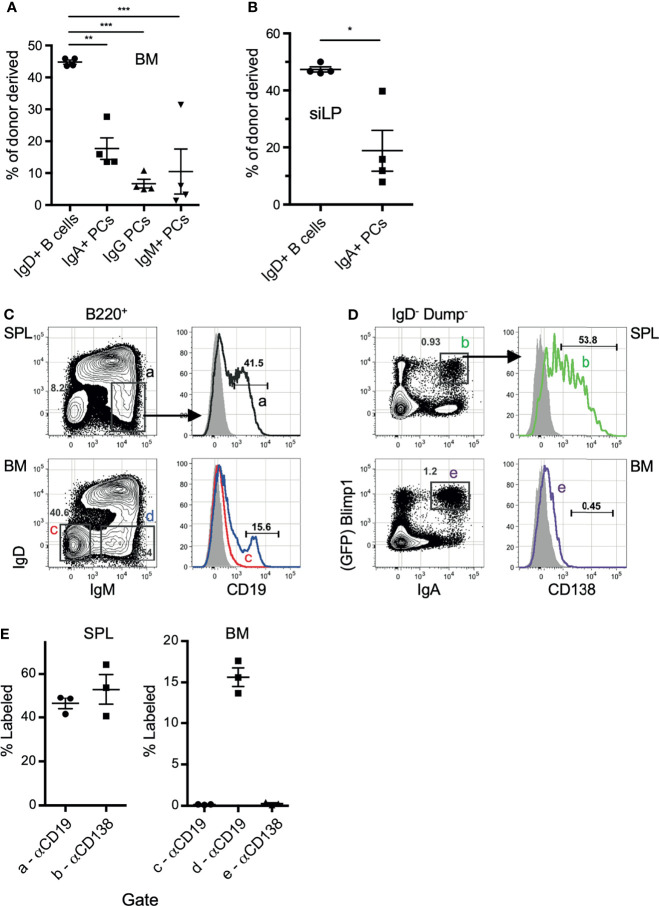
IgA^+^ plasma cells show little evidence of recirculation. **(A, B)** C57BL/6 (CD45.2) and B6.CD45.1 adult females were connected by parabiosis and were monitored for blood sharing. After 17 days, mice were euthanized and the frequency of donor plasma cells in BM **(A)** and siLP **(B)** was determined by flow cytometry using CD45.1/CD45.2. **(C, D)** B6.Blimp1^+/GFP^ adults were given an intravenous (i.v.) injection of PE-anti-CD19 **(C)** or PE-anti-CD138 antibodies **(D)** and sacrificed two minutes later. BM cells were stained with the indicated antibodies and 2 x 10^6^ events were collected to identify cells bound by either PE-labeled antibody *in situ*. Grey histograms represent the B cells from naïve control mice. **(E)** Frequency of cells bound by PE-anti-CD138 antibodies in the indicated tissue. Each symbol represents an individual mouse; red lines and error bars indicate the mean and SEM for each group. Gates are shown in **(C)**. Representative of 2 independent experiments with 4 mice per group. * indicates p < 0.05, ** indicates p < 0.01, and *** indicates p < 0.001.

**Figure 3:**
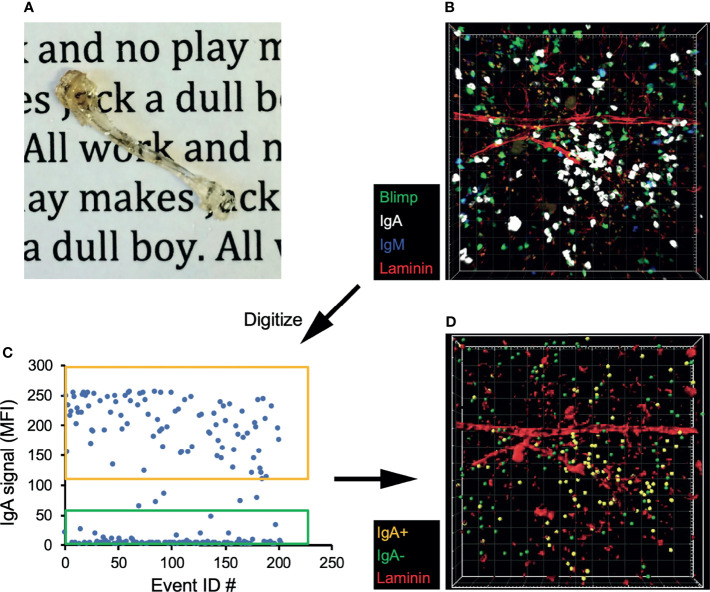
IgA^+^ BM plasma cells reside in the perivascular plasma cell niche. **(A)** Tibia from a B6.Blimp1^+/GFP^ mouse after fixation and clearing. **(B)** 3D confocal image of a cleared tibia from a B6.Blimp1^+/GFP^ female stained with CF568-anti-Laminin, CF647 anti-IgA and BV421 anti-IgM. **(C)** Using Imaris software, Blimp-GFP^+^ plasma cells were identified and sorted as IgA^+^ or IgA^-^ based on their mean fluorescent intensity (MFI) values for IgA. **(D)** A model of the original 3D image displaying the sorted IgA^+^ (gold) and IgA^-^ (green) plasma cells overlaid with Laminin^+^ vasculature.

We next sought to confirm that IgA-secreting BM cells are not a circulating population, but located within the BM parenchyma long-lived plasma cell niche. To address this, we took multiple approaches, beginning with performing parabiosis using mice distinguished by congenic markers. The mice were joined for 17 days, then flow cytometric analysis of BM and siLP plasma cells was performed to determine the percentage of donor cells that contributed to the standing plasma cell pool. As expected, we found that naïve IgD^+^ B cells were nearly 50% donor-derived in both the BM and siLP, indicating that parabiosis resulted in adequate blood sharing (**Figures 2A, B** **)**. In contrast, the IgA^+^ plasma cells in both the BM and siLP were <20% donor-derived, indicating that they were not in constant recirculation like their naïve B cell counterparts. The IgG^+^ BM plasma cell pool was found to be <10% donor derived. The difference between the frequency of donor-derived IgA and IgG plasma cells may be due to a real difference in recirculation or alternatively a reflection of a higher frequency of new IgA^+^ plasma cell generation following surgery and prophylactic antibiotic treatment. Therefore, we sought to support these findings by utilizing additional methods, including an approach wherein cells located in BM sinusoids are labeled two minutes following intravenous (i.v.) injection with PE-labeled antibodies (38). Confirming their parenchymal residency, BM IgA^+^ plasma cells were not labeled by this approach, whereas immature splenic and BM B cells (IgM^+^ IgD^-^) and splenic plasma cells were readily labeled following i.v. injection with anti-CD19 or anti-CD138, respectively (**Figures 2C–E**). Additionally, recent work suggests that BM plasma cells preferentially localize in perivascular regions in close proximity to HSCs and regulatory T cells (39). To determine the location of IgA^+^ relative to IgA^-^ plasma cells in the BM, we performed whole bone tissue clearing (20) to facilitate 3-dimensional confocal imaging of intact bones from B6.Blimp1^GFP^ transgenic mice along with antibody staining for IgA (**Figures 3A, B** **)**. Coordinates of IgA^+/-^ plasma cells were mapped by 3-D digital reconstruction, resulting in the detection of IgA^+^ plasma cells in perivascular regions of the BM parenchyma similar to IgA^-^ counterparts (**Figures 3C, D** **)**. These results suggest there is a low level of IgA^+^ plasma cell recirculation or IgA^+^ plasma cells are more frequently generated and deposited in the BM during homeostasis. Overall, these data suggest that IgA^+^ plasma cells are non-transient residents of the BM and siLP tissues.

### Antibody Repertoires and Gene Expression Signatures Are Shared by BM and siLP Plasma Cells

**Figure 4:**
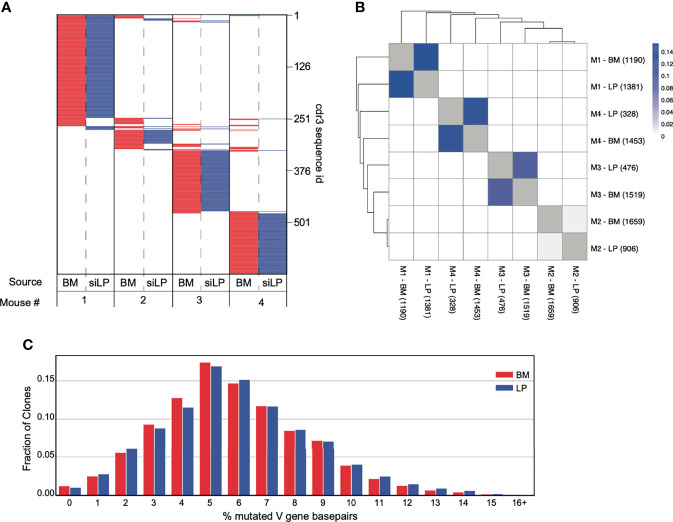
Significant overlap of clones between IgA^+^ plasma cells from the BM and siLP. V_H_D_H_J_H_ rearrangements were amplified from cDNA prepared from sorted siLP and BM IgA^+^ plasma cells and sequenced on an Illumina MiSeq. Sequences were quality filtered and collapsed into clones as described in materials and methods. Clones were filtered based on size such that clones with a copy number less than 50% of the mean copy number frequency (50% mcf) within the subject were excluded. **(A)** Representation of CDR3s overlapping in at least two IgA^+^ biological samples. Each horizontal line represents a unique CDR3 sequence, the presence of red (bone marrow) or blue (siLP) indicates an overlapping sequence was detected in a sample and white indicates the absence of that CDR3 in a given sample. The total number of clones is shown on the right side of the plot. **(B)** Jaccard index was calculated to compare mouse clonal overlap of BM and siLP between vs. within each mouse. Each clone counts once, disregarding clone size. Numbers in parenthesis are the number of clones included in this study for a given sample. **(C)** Somatic hypermutation percentage histogram of IgA^+^ clones stratified by BM and LP. The degree of SHM within each clone was averaged across each unique sequence and weighted by the number of copies.

Next, we sequenced productive V_H_D_H_J_H_-Cα rearrangements from IgA^+^ plasma cells in the BM and siLP of individual mice to evaluate potential clonal relationships between these cells. We then quantified frequencies of shared V_H_D_H_J_H_ sequences, including shared third complementarity determining regions (CDR3) in each sample from each individual. Consistent with recent work (22), the clonal composition of IgA-secreting cells was notably distinct for each mouse (**Figure 4A**). However, in each of two separate experiments we observed measurable clonal overlap for siLP and BM IgA^+^ plasma cells within each animal (**Figures 4A, B** **)**. Furthermore, the expressed V_H_D_H_J_H_-Cα repertoires for BM and siLP plasma cells within each mouse were both highly enriched for clones that had undergone somatic hypermutation (SHM). Additionally, the mutation rate was similar between both tissues (**Figure 4C**), again indicative of T-cell dependent GC-associated antibody responses.

### Isotype-Dependent Gene Transcription Profiles

**Figure 5:**
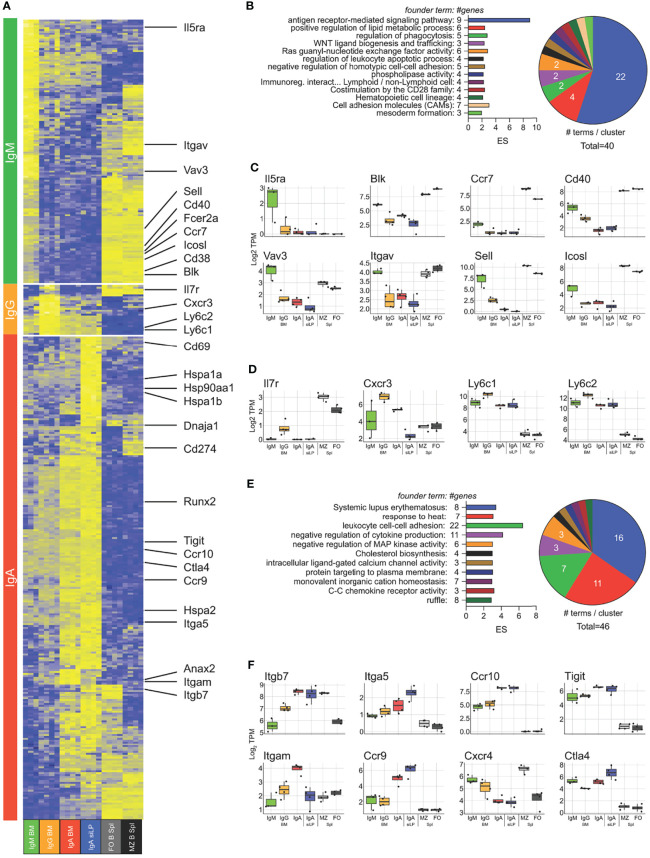
Isotype-specific gene expression crosses tissue boundaries. RNA-seq was performed on isotype-specific plasma cells twice sorted from BM and siLP of B6.Blimp1^+/GFP^ mice as in **Figure 1A**. **(A)** Genes upregulated in each isotype over remaining isotypes by at least 2-fold with an adjusted P value <.01 are shown with focus genes highlighted. IgA specific genes are the union of each organ-specific genelist. Lists of differentially-expressed genes are found in **Supplementary Table 1**. **(B)** Gene ontology cluster enrichment analysis for IgM plasma cell-specific genes is shown. For each cluster, the founder term was chosen as the term with the most significant Bonferroni step down adjusted P value. The number of genes for the cluster term is shown following the term name. The enrichment score [-log_10_(adj-P)] is shown as bar length while the pie slice indicates number of enriched terms grouped in each cluster. **(C, D)** The log_2_ TPM magnitude of expression of selected IgM^+^ and IgG^+^ plasma cell-specific genes, respectively, is shown as the mean (bar), 75% c.i. (box), 95% c.i. (whisker) and individual animal data points (jitter). **(E)** Gene ontology cluster enrichment of IgA-specific genes from both BM and LP is displayed as in **(B)**. **(F)** Log_2_ TPM magnitude of expression of IgA^+^ plasma cell-specific is displayed as in **(C)**. Differential gene expression in **(A)** was determined by empirical Bayes method with Benjamini and Hochberg correction for multiple comparisons.

In order to examine isotype-dependent and -independent gene expression in plasma cells, we performed RNA-seq on cDNA prepared from twice sorted IgM^+^, IgA^+^ and IgM/A^-^ BM plasma cells, as well as IgA^+^ and IgA^-/-^ (Blimp^GFP+^) siLP plasma cells. For comparison we also sorted splenic follicular- and marginal zone-B cells. Isotype specific plasma cell samples clustered strongly by isotype with IgA+ plasma cells clustering closer regardless of tissue and IgA^-/-^ siLP plasma cells clustering closest to IgM/A^-^ BM plasma cells (**Figure S2A**). To validate our double sorting method, we verified that marker genes of common contaminating cells were not expressed in our plasma cell groups (**Figure S2B**). We then determined an isotype-independent plasma cell signature by identifying all genes upregulated at least 3-fold over the B cell populations in all plasma cell subsets, yielding a total of 308 genes (**Figure S2C**). Comparing our results to the plasma cell signature published in Shi et al. (40) revealed several differences that are likely the result of varying frequencies of IgA plasma cells resulting from likely microbiome differences between facilities (7) and our use of both marginal zone (41) and follicular B cells instead of follicular B cells alone to develop our plasma cell signature (**Figure S2C**). Functional enrichment analysis of these genes showed more than half of the enriched GO terms to be related to ER function (**Figure S2D**). Given how the composition of isotypes varied the expression of non-immunoglobulin plasma cell signature genes, we sought to analyze isotype-specific gene expression between all plasma cell subsets, without respect to immunoglobulin itself. We identified 1757 genes significantly differentially expressed between all plasma cell subsets. Hierarchical clustering of these genes showed that there was a strong association between the IgA^+^ plasma cells in the BM and siLP, with genes coregulated in these samples being enriched for cytoplasmic heat shock proteins and cholesterol homeostasis (**Figures S2C, E–G**). To better determine isotype specific gene expression, we prepared contrast matrices to determine genes differentially upregulated in each isotype versus all other isotypes with IgA-specific gene expression being defined as genes upregulated in either BM or siLP IgA^+^ plasma cells over IgM^+^ and IgG^+^ plasma cells (**Figure 5A**). IgM^+^ plasma cell genes were functionally related to B cell activation and BCR signaling (**Figures 5A–C**). IgG-specific genes were few but included the IL-7 receptor (*Il7ra)* and Cxcr3 (**Figures 5A, D** **)**, consistent with the association of T-bet/Cxcr3 expression with IgG2c (42). IgA-specific genes were the largest group and included genes functionally related to heat shock, negative regulation of signaling, and a large group of histone genes associated with SLE (**Figures 5A, E, F** **)** (43). Interestingly there were genes related to cell positioning that were shared between the siLP and BM IgA^+^ samples as compared with other BM plasma cell isotypes, such as higher expression of Ccr9 and Ccr10 and lower expression of surface CXCR4 which was confirmed by flow cytometry (**Figures 5F**, **S3A**). These results indicate that BM IgA^+^ plasma cells use a different program to home to the BM than other isotypes. Further characterization of these cells by flow cytometry revealed that BM IgA^+^ plasma cells were negative for the gut homing chemokine receptor CCR9, despite having high levels of RNA transcripts and surface expression of the integrin heterodimer α_4_β_7_ (**Figure S3A**). To further examine IgA^+^ plasma cell gene expression as it relates to tissue, we identified genes that were up or down regulated in common between the siLP and BM samples (**Figures S3B, C**). Upregulated genes were enriched for functions associated with intestinal IgA production, while downregulated genes were enriched for inflammatory mediator functions.

**Figure 6:**
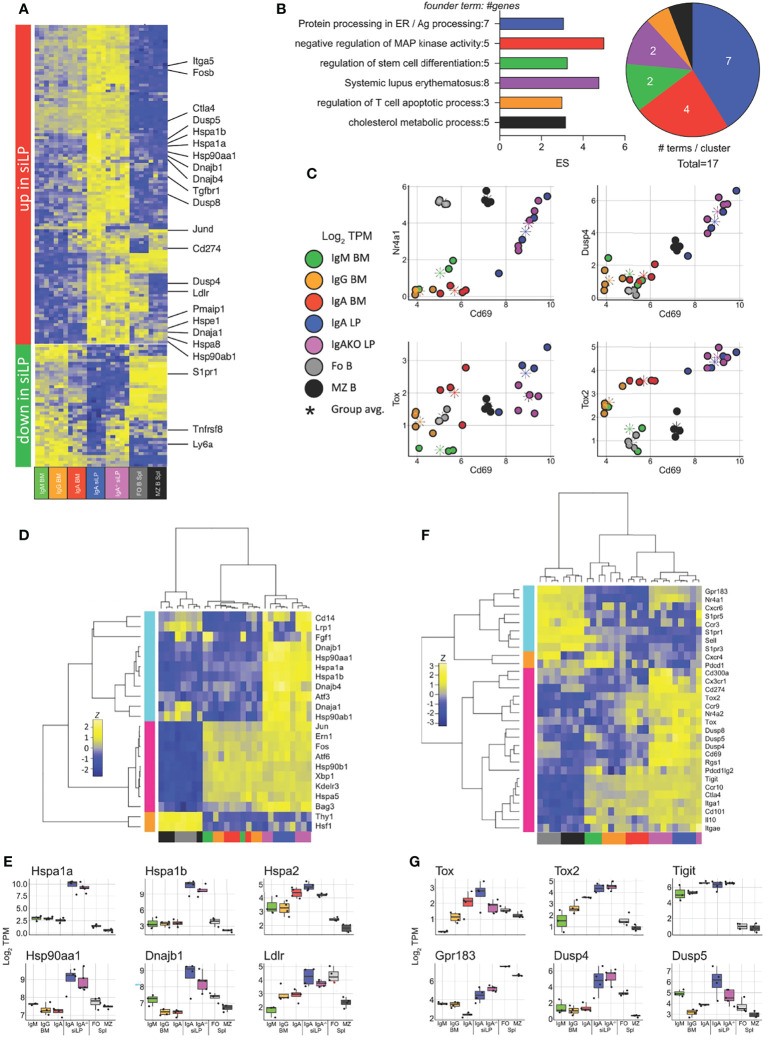
Lamina propria plasma cells express a unique transcriptional program in response to tissue-specific signals. RNA-seq was performed as in **Figure 5** with the addition of plasma cells sorted from the siLP of B6.Blimp1^+/GFP^.IgA^-/-^ mice. **(A)** Genes differentially up and down regulated in IgA^+^ siLP plasma cells compared with all BM plasma cell subsets by at least 2-fold with an adjusted P value <.01 are shown with focus genes highlighted. Complete lists of differential genes are found in **Supplementary Table 1**. **(B)** Gene ontology cluster enrichment analysis for IgA^+^ siLP plasma cell-specific genes is shown. For each cluster, the founder term was chosen as the term with most significant Bonferroni step down adjusted P value. The number of genes for the cluster term is shown following the term name. The enrichment score [-log_10_(adj-P)] is shown as bar length while the pie slice indicates number of enriched terms grouped in each cluster. **(C)** Scatter plots of all groups and all animals for the indicated two gene comparisons is shown as log_2_ TPM expression (colored circles). The colored asterisk indicates the average for each displayed group. **(D)** plasma cell-related UPR gene and heat shock gene expression is shown. Genes and samples were allowed to cluster freely by Pearson and Spearman correlation, respectively. **(E)** Individual log_2_ TPM gene expression magnitude is shown for selected genes as the mean (bar), 75% c.i. (box), 95% c.i. (whisker), and individual values for each animal (jitter). **(F)** Genes contributing to tissue residency, central lymphoid residency and effector cell exhaustion were used to cluster samples as in **(D)**. **(G)** Individual log_2_ TPM gene expression magnitude is shown for selected genes as in **(E)**. Differential gene expression in **(A)** was determined by empirical Bayes method with Benjamini and Hochberg correction for multiple comparisons.

We then sought to determine what tissue-driven gene expression differences exist between the siLP and BM PC compartments. To isolate the effect of location in the siLP from IgA expression, we compared the plasma cell samples described above with siLP plasma cells from IgA^-/-^ mice. The isotypes present in siLP plasma cells of the IgA^-/-^ mice consisted primarily of IgG2b (61%), IgG1 (23.5%), and IgM (12%) (**Figure S3D**). Genes downregulated specifically in siLP samples included those required for emigration from secondary lymphoid structures, such as S1pr1, and genes that modulate lymphoid activation, such as the gene encoding CD30 (*Tnfrsf8*) (**Figure 6A**). Genes upregulated in siLP samples were enriched for functions important in protein processing in the cytosol such as the HSP70 family members Hspa1a and Hspa1b, negative regulation of map kinase signaling such as Dusp4 and Dusp8, and several inhibitory markers often associated with T cell exhaustion and tissue residency, such as Ctla4, PD-L1 (*Cd274*) and CD69 (**Figures 6A, B** **)**. We then examined correlation of the activation marker CD69 and several activation-induced inhibitory modulators to infer environmental cues affecting gene expression in siLP plasma cells (**Figure 6C**). We observed strong correlation between CD69 expression and both Nur-77 (*Nr4a1*) and Dusp4 in siLP samples. We additionally observed correlation between expression of CD69 and the related transcriptional repressors Tox and Tox2 in siLP samples (**Figure 6C**). Given that heat shock protein family genes were among the most upregulated genes in siLP plasma cell samples, we prepared a shortlist of genes containing both canonical ER-located UPR-associated genes and cytoplasmic heat shock protein genes implicated in antigen processing, and allowed all samples to cluster freely based on the expression of these genes. This approach yielded three groups with no respect to isotype but instead samples grouped strongly into B cells, BM plasma cell samples and siLP plasma cell samples (**Figures 6D, E** **)**. This result indicated that cytoplasmic heatshock (HSP40, HSP70) family member gene expression was tissue-restricted to siLP plasma cells and is not carried over into BM IgA^+^ plasma cells, while as expected ER-associated HSPs were common to all plasma cell groups. Finally, we sought to determine if siLP plasma cells displayed a tissue residency phenotype. We prepared a list of genes including markers of tissue residency, secondary lymphoid (central) homing and several negative regulators of activation or exhaustion markers. These genes grouped the siLP samples closest together, with siLP samples expressing many of the residency program genes and exhaustion genes (**Figures 6F, G** **)**. Interestingly, these genes also segregated BM plasma cells by isotype with IgA^+^ BM plasma cells being more closely related to siLP plasma cells by expression of these genes indicating that these gene expression programs may be more closely tied to the IgA isotype.

## Discussion

Our work demonstrates that IgA^+^ plasma cells are long-lived residents of both the BM and siLP. This contrasts with the traditional view of siLP plasma cells as short-lived, largely T-independent effectors that are generally restricted to mucosal tissues. We show that IgA^+^ plasma cells in the siLP and BM are related both clonally and transcriptionally. These cells have been previously shown to be, at least in part, induced by and specific to commensal bacteria. Thus, providing an IgA^+^ plasma cell reservoir for long-term protection from barrier disruption or transient changes in gut microbiome. In spite of the high degree of similarity between IgA^+^ plasma cells in the siLP and BM, we were able to identify a tissue specific transcriptional gene expression pattern in siLP plasma cells with hallmarks of tissue resident T cells and uniquely high expression of cytoplasmic heat shock proteins. Therefore, while the expression of particular Ig heavy chain isotypes correlates with distinct gene expression signatures (15), plasma cell gene expression is also influenced by tissue localization, independent of heavy chain expression.

The role of long- and short-lived IgA^+^ plasma cells in the siLP is generally believed to be the maintenance of homeostasis and to respond to changing components of the microbiome. However, the need for long-lived IgA^+^ plasma cells in systemic sites such as the BM has not been fully explored, and it has only been recently described that long-lived IgA^+^ plasma cells can be induced in the BM in the context of immunization. Using carbon dating Landsverk et al. described IgA^+^ plasma cells within human gut tissue that appeared to persist for decades (3), but without single cell analyses it remained unclear whether longevity is a common feature for gut-resident plasma cells. Our data demonstrate that the vast majority of IgA^+^ plasma cells are long-lived regardless of the tissue of residence. However, while BrdU labeling reveals that the majority of IgA^+^ plasma cells are long-lived, we also observe that roughly 20% of the population is rapidly labeled and that population size remains stable. These data suggest that at least a portion of the IgA^+^ plasma cells in both the bone marrow and siLP are indeed turning over frequently. Beyond that, we find that while BM and siLP IgA^+^ plasma cells are clonally related, despite the absence of routine migration of cells to and from the gut and BM. Furthermore, despite expression of different chemokine receptors and integrins, the IgA^+^ plasma cells in the BM occupy similar niches compared to IgG^+^ and IgM^+^ plasma cells. If IgA^+^ plasma cells are capable of providing long-lived protection, then their induction is potentially of use in vaccine strategies or in the context of probiotics. The unique functional ability of IgA to act in an anti-inflammatory manner could prove beneficial in some contexts and undesirable in others. Recent work has demonstrated unique roles of IgA^+^ plasma cells in the siLP and other contexts such as multiple sclerosis. The implications of the work demonstrated here are that IgA^+^ plasma cells can have long-term impacts on health in a variety of contexts.

Interestingly, the IgA^+^ plasma cells in the BM expressed low levels of CXCR4 on their surface and had low mRNA expression. Studies in CXCR4^-/-^ animals have demonstrated dramatic reductions in BM plasma cell frequencies and no other known chemokine receptors have been implicated in plasma cell BM homing. Our findings suggest an alternative mechanism for IgA^+^ plasma cell migration to the BM. Furthermore, IgA^+^ plasma cells in the BM retain mRNA expression of gut associated chemokine receptors CCR9 and CCR10, as well as the mucosal retention integrin α_4_β_7_. The exact mechanism used by IgA^+^ plasma cell for BM transit is still under investigation and our findings point to gene expression differences in S_1_PR_1_ and integrin α_5_ as potential players. However, transcriptomics only paints a partial picture and this is highlighted by high Ccr9 transcripts in bone marrow IgA^+^ plasma cells, but low CCR9 surface protein levels as demonstrated by flow cytometry. Intriguing findings associating siLP IgA^+^ plasma cells with canonical tissue residency gene expression profiles defined from work with T cell subsets are also targets of further expansion of this line of experimentation.

Heterogeneity of plasma cell gene expression based on isotype has been previously described by Price et al. Our work expands on their findings in several ways, first by examining steady state bone marrow isotype specific plasma cells, and importantly, by the addition of IgA^+^ plasma cells from the siLP providing us the ability to identify tissue-specific gene expression patterns. Additionally, we demonstrate that sorting plasma cells by isotype can be performed without the need of fixation and permeabilization. This opens the door to further functional studies of plasma cells based on isotype. Our RNA-seq dataset has slight variations from Price et al. that may be related to the methodology used. The work herein was not performed in an antigen specific manner, but surface staining of plasma cells using antigen-specific probes without the need for fixation/permeabilization is also possible. Past work from our laboratory suggests that isotype is a much larger source of plasma cell heterogeneity than found comparing long- or short-lived plasma cell irrespective of isotype. It is unclear if sorting BM plasma cells by isotype and B220 expression would result in the identification of significant functional differences based on longevity and isotype combined.

This work reiterates that isotype-driven transcriptional programs play a key role in plasma cell migration and function. Our data expand on this by demonstrating tissue-specific plasma cell gene transcription that has implications for strategies to induce systemic IgA. Additionally, the long-lived nature of IgA^+^ plasma cells in the siLP and BM suggest that induction of IgA could be advantageous for durable immunity.

## Data Availability Statement

The datasets presented in this study can be found in online repositories. The names of the repository/repositories and accession number(s) can be found below: NCBI: PRJNA783313 and GSE189410.

## Ethics Statement

The animal study was reviewed and approved by Office of Regulatory Affairs Institutional Animal Care and Use Committee. Written informed consent was obtained from the owners for the participation of their animals in this study.

## Author Contributions

JW, BG, and DA designed experiments and wrote the manuscript. BG performed RNA-seq bioinformatics analyses. JW, BG, DA, RR, and SR performed experiments. WM, AR, and EL executed the sequencing and bioinformatics analysis of antibody repertoires. All authors contributed to the article and approved the submitted version.

## Funding

This work was supported by National Institutes of Health (NIH) grants R01-AI139123 and R01-AI154932 to DA and F32-AI114089 to JW. EL was supported by NIH grant P30-CA016520, BG by NIH training grant T32CA009140, and RR by NIH training grant T32-AI055428.

## Conflict of Interest

The authors declare that the research was conducted in the absence of any commercial or financial relationships that could be construed as a potential conflict of interest.

## Publisher’s Note

All claims expressed in this article are solely those of the authors and do not necessarily represent those of their affiliated organizations, or those of the publisher, the editors and the reviewers. Any product that may be evaluated in this article, or claim that may be made by its manufacturer, is not guaranteed or endorsed by the publisher.
