# Supplementary material for: IgA Plasma Cells Are Long-Lived Residents of Gut and Bone Marrow That Express Isotype- and Tissue-Specific Gene Expression Patterns
Source: Front Immunol. 2021 Dec 24;12:791095. doi: 10.3389/fimmu.2021.791095 (PMC8739487; doi:10.3389/fimmu.2021.791095)
Supplement: Supplementary file 1 [file DataSheet_1.pdf]

Supplementary Information

Supplementary Figure 1: this file

Supplementary Figure 2: this file

Supplementary Figure 3: this file

Supplementary Table 1: Supplementary_Table_1.xlsx

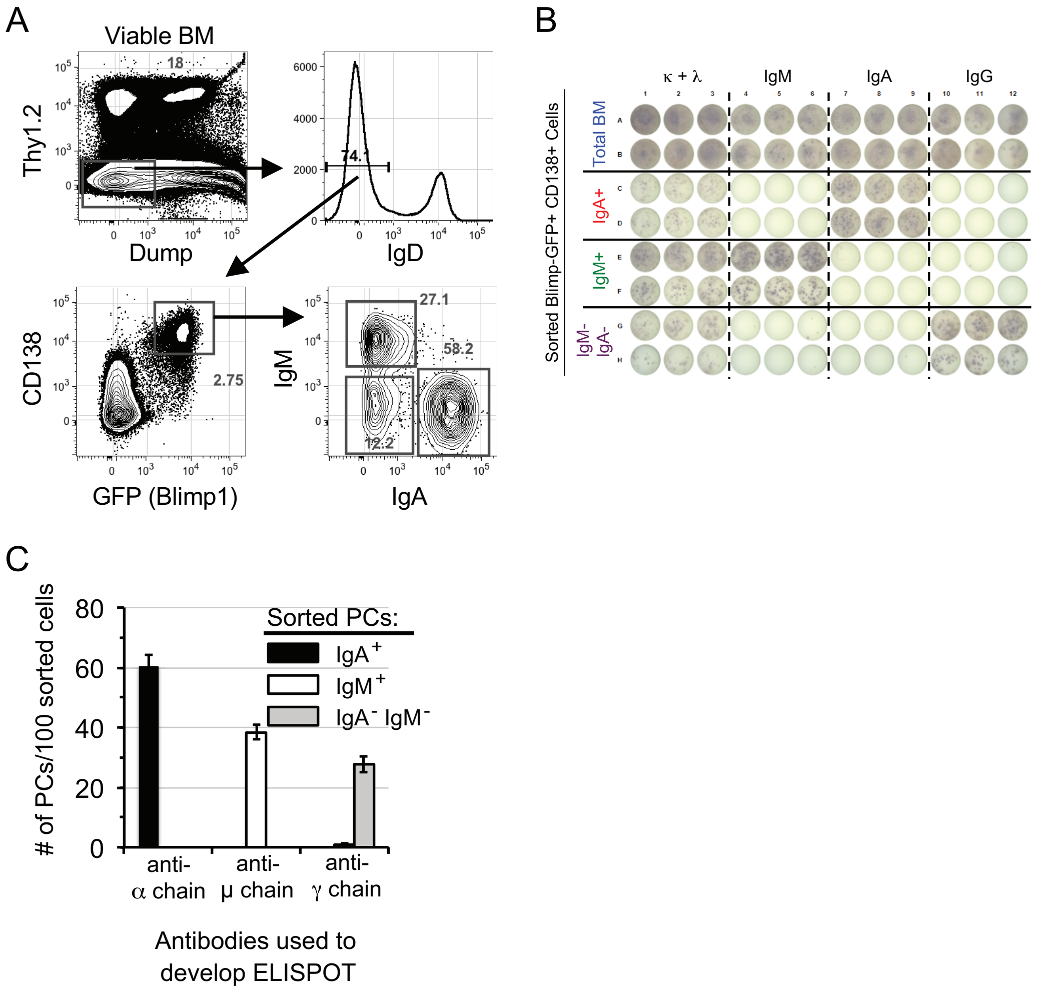

Supplementary Figure 1: plasma cells can be sorted by isotype without fixation. (A) Viable BM plasma cells were detected by flow cytometry and defined as Thy1.2-, Dump- (CD4, CD8, F4/80, Ter119), IgD$^-$, CD138$^+$, Blimp-$^{GFP+}$. Using antibodies to detect IgA and IgM we sorted the IgA$^+$ IgM$^-$, IgM$^+$, IgA$^-$, and IgA$^-$ IgM$^-$ populations. (B-C) Each subset from (A) was incubated on ELISpot plates coated with anti-Ig(H+L), then incubated overnight at 37° C in a 5% CO_2 incubator. Each sorted subset was then stained with anti-IgG, anti-IgM, anti-IgA, or anti-κ+λ (total Ig). (C) Graphical representation of data in (B).

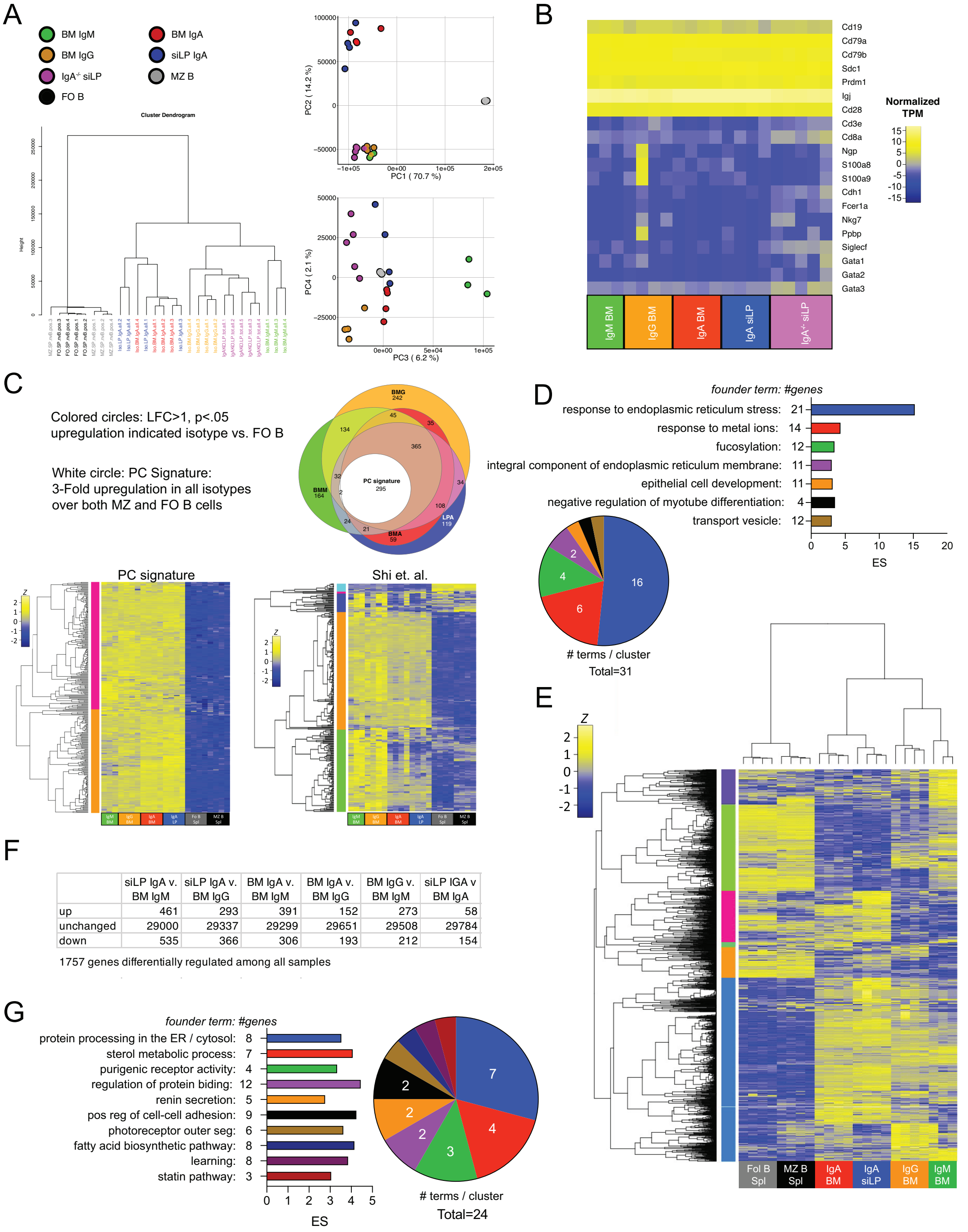

	siLP IgA v. BM IgM	siLP IgA v. BM IgG	BM IgA v. BM IgM	BM IgA v. BM IgG	BM IgG v. BM IgM	siLP IGA v BM IgA
up	461	293	391	152	273	58
unchanged	29000	29337	29299	29651	29508	29784
down	535	366	306	193	212	154

1757 genes differentially regulated among all samples

Supplementary Figure 2

Supplementary Figure 2: Isotype-specific gene expression crosses tissue boundaries. (A) Hierarchical clustering of all samples described in Figure 1 by all transcripts is shown (left) with

principal component analysis displaying first four components (right). (B) B cell, plasma cell and other common marker genes for possible contaminating cell types are shown as a heatmap of normalized TPM. (C) Genes upregulated 2-fold in each isotype specific plasma cell group over follicular B cells (colored circles) as well as plasma cell signature genes, upregulated by 3-fold in all isotypes over both FO and MZ B samples (white circle) are displayed as a Euler plot (top). Plasma cell signature gene expression is compared (left) to expression of Shi et. al. gene expression (right) for all samples described in Figure 1. (D) Gene ontology cluster enrichment of plasma cell signature genes described in (C) is shown. For each cluster, the founder term was chosen as the term with most significant Bonferroni step down adjusted P value. The number of genes for the cluster term is shown following the term name. The enrichment score [$-\log_{10}$(adj-P)] is shown as bar length while the pie slice indicates number of enriched terms grouped in each cluster. (E, F) Contrasts between siLP IgA^+ plasma cell and all BM plasma cell samples defined 1757 differentially up and down regulated genes by at least 2-fold with an adjusted P value <.01 were used to cluster all genes and samples by Pearson and Spearman correlations respectively (E) and are enumerated by contrast matrix coefficient. (F) Genes upregulated similarly in IgA^+ plasma regardless of tissue in the blue cluster from (E) were subjected to gene ontology cluster enrichment analysis which is displayed as in (D). Differential expression in (C, E, F) determined by empirical Bayes method with Benjamini and Hochberg correction for multiple comparisons.

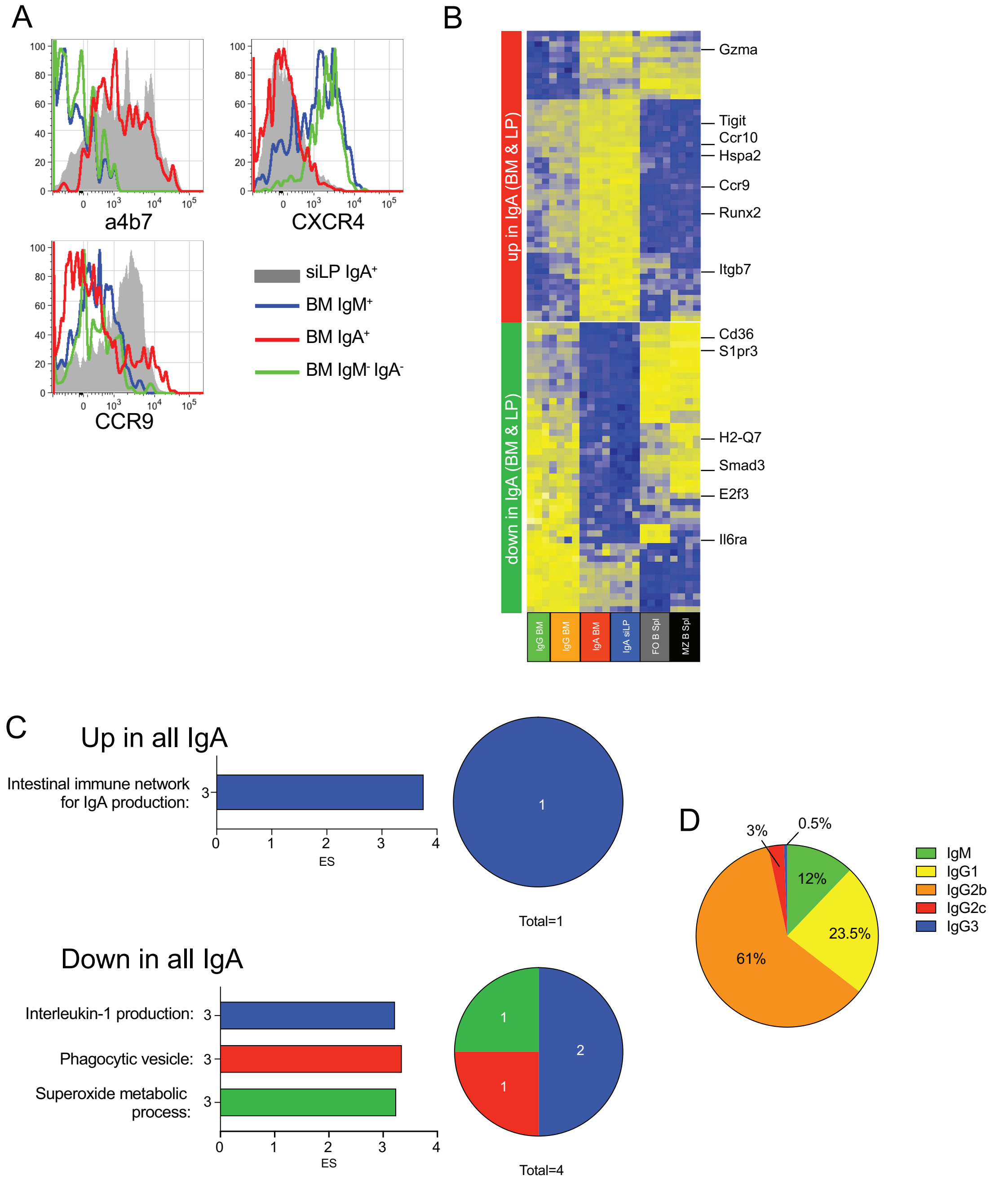

Supplementary Figure 3: Lamina propria plasma cells express a unique transcriptional program in response to tissue specific signals. (A) BM and siLP cells were isolated and stained to detect plasma cells by flow cytometry as in Figure S1. Antibodies specific to the $\alpha_4\beta_7$ heterodimer, CXCR4, and CCR9 were used to assess their expression levels on each isotype of

Dump$^-$, IgD$^-$, CD138$^+$, Blimp-GFP$^+$ cells. Shown are representative images of 4 mice per group. (B) Expression of genes significantly up and downregulated in both BM and siLP IgA$^+$ plasma cell groups by at least 2-fold with an adjusted P value <.01 are shown as z score across each row with focus genes highlighted. (C) Gene ontology cluster enrichment of genes described in (B) is shown. For each cluster, the founder term was chosen as the term with most significant Bonferroni step down adjusted P value. The number of genes for the cluster term is shown following the term name. The enrichment score [$-\log_{10}$(adj-P)] is shown as bar length while the pie slice indicates number of enriched terms grouped in each cluster. (D) ELISpot was used to determine the isotypes present in the siLP of IgA$^{-/-}$ mice. For each isotype 20,000 cells were plated and cultured overnight on plates coated with anti-Ig(H+L). Spots were detected using isotype-specific antibodies for IgM, IgG1, IgG2b, IgG2c, and IgG3. Shown is the frequency of total number of spots averaged from 3 mice. Differential expression in (B) determined by empirical Bayes method with Benjamini and Hochberg correction for multiple comparisons
